# Ultrasound Versus Fluoroscopy for Cervical Medial Branch Injections at C3–C6: A Single-Center Retrospective Cohort Study

**DOI:** 10.3390/diagnostics16040592

**Published:** 2026-02-16

**Authors:** Cagatay Kucukbingoz, Ahmet Yılmaz

**Affiliations:** Department of Algology, Adana City Training and Research Hospital, 01370 Adana, Turkey; dr.ahmetyilmaz27@gmail.com

**Keywords:** cervical facet joint, chronic neck pain, fluoroscopy, single-center, non-inferiority, radiation safety, radiofrequency ablation, ultrasound guidance

## Abstract

**Objective:** Cervical facet joints are prominent sources of chronic neck pain. This single-center retrospective cohort study compared ultrasound (US)-guided and fluoroscopy (FL)-guided cervical medial branch procedures at the C3–C6 levels in terms of technical success and tested for non-inferiority (NI). **Methods:** Between March 2022 and July 2025, 224 procedures performed at the Adana City Training and Research Hospital, Department of Algology (US, *n* = 104; FL, *n* = 120), were analyzed. The primary endpoint was technical success. Secondary endpoints included procedure duration; complications; pain intensity, measured using the Visual Analog Scale (VAS); functional outcomes, assessed with the Neck Disability Index (NDI) at 1, 3, 6, and 12 months; and radiation exposure in the FL group. Propensity score weighting was applied to reduce confounding. The NI margin for technical success was set at −5 percentage points, with α = 0.025. **Results:** The technical success rates were similar (US: 90.4%, FL: 90.8%; difference: −0.4%, 95% CI: −8.1 to 7.2), but non-inferiority was not statistically achieved (power ≈ 72%). Procedure time was significantly shorter in the ultrasound-guided group compared with the fluoroscopy-guided group (mean difference −5.2 min; 95% CI: −7.5 to −2.9; *p* < 0.001). No major complications occurred. Both groups demonstrated sustained improvement over 12 months, with VAS scores decreasing from 7.4 at baseline to 4.0 at 12 months and NDI scores decreasing from 41.3 to 29.2. No statistically significant between-group differences were observed for pain intensity (VAS) at any follow-up time point (all *p* > 0.05). Similarly, functional outcomes (NDI) did not differ significantly between the ultrasound- and fluoroscopy-guided groups throughout follow-up (all *p* > 0.05). Exploratory analyses using minimum clinically important difference (MCID) thresholds supported the clinical non-inferiority of US. In the FL group, the mean fluoroscopy time was 2.28 min with low radiation doses. **Conclusions:** Although NI was not statistically confirmed for technical success, US achieved comparable outcomes to FL with shorter procedure times and without radiation exposure. Both modalities provided similar long-term pain relief and functional improvement. US can be considered a safe and effective alternative in experienced hands, but larger multicenter prospective NI trials are needed to confirm these findings.

## 1. Introduction

Neck pain is a prevalent condition that can substantially reduce quality of life and necessitates the extensive utilization of healthcare services. Among the various structures contributing to cervical pain, facet joints are recognized as a significant source. Clinical series based on controlled blocks have consistently demonstrated that cervical facet joints account for a substantial proportion of facet-related pain. Current multidisciplinary guidelines recommend diagnostic and therapeutic medial branch blocks in appropriately selected patients, with Radiofrequency Ablation (RFA) reserved for refractory cases [1,2,3,4].

Contemporary epidemiological estimates underscore the scale of this problem. The Global Burden of Disease (GBD) 2021 analysis estimated that neck pain affected approximately 203 million people globally in 2020, with Western Europe alone accounting for around 17.1 million prevalent cases [1,2]. In parallel with this high prevalence, cervical facet interventions are performed at substantial volume; multispecialty consensus guidelines highlight the widespread use of cervical medial branch blocks and radiofrequency techniques for facet-mediated neck pain and note increasing utilization in routine practice in some health systems [5].

Traditionally, fluoroscopy (FL) has been considered the reference standard for cervical medial branch procedures due to its ability to delineate bony landmarks and provide multiplanar verification. However, it exposes both patients and healthcare providers to ionizing radiation. In contrast, ultrasound (US) enables real-time visualization of soft tissues and vascular structures, eliminates radiation exposure, and may facilitate needle guidance. Despite these advantages, US image quality is operator-dependent and may be limited by the acoustic window. Comparative studies suggest that US achieves similar technical and clinical success rates to FL in cervical interventions while offering efficiency advantages, such as reduced procedure duration and fewer needle passes. This has been demonstrated in randomized studies targeting the C7 medial branch and the third occipital nerve, as well as in cohort studies involving cervical medial branch blocks [5,6,7].

Recent advances in US technology further support its expanding role in interventional pain practice. Improvements in transducer resolution, image-processing techniques (including compound and tissue harmonic imaging), and Doppler capabilities have enhanced visualization of cervical musculoskeletal and neurovascular structures. In parallel, the increasing availability of portable, high-performance US systems and the growth of operator expertise—supported by structured training programs and consensus guidelines—have facilitated broader adoption of US-guided cervical spine interventions in routine clinical settings [5,8,9,10,11].

Despite these promising findings, important evidence gaps remain. Existing randomized trials have primarily focused on C7 or the third occipital nerve, with limited direct comparisons at the C3–C6 levels within a non-inferiority (NI) framework. Furthermore, few studies have simultaneously reported key procedural outcomes such as technical success, procedure time, complication rates according to the Cardiovascular and Interventional Radiological Society of Europe (CIRSE) classification, patient-reported outcomes including the Visual Analog Scale (VAS) and Neck Disability Index (NDI), and radiation metrics in the FL arm. Additionally, there is a lack of real-world single-center evidence exploring potential effect modifiers across clinically relevant subgroups, such as Body Mass Index (BMI) and gender [5].

Based on these gaps, the present single-center retrospective cohort study was designed to compare US- and FL-guided cervical medial branch block/ablation procedures at the C3–C6 levels. The primary endpoint was the technical success rate, which was tested for NI using a margin of −5 percentage points and a one-sided significance level of α = 0.025. Secondary endpoints included procedure duration; complications graded according to CIRSE criteria; pain reduction, measured by the VAS; functional improvement, assessed using the NDI at predefined follow-up intervals (1, 3, and 6 months); and radiation exposure metrics in the FL group (fluoroscopy time, dose–area product [DAP], and air kerma). Subgroup analyses were prespecified according to BMI and gender. The study design aligns with current international methodological and reporting recommendations [12,13].

We hypothesized that ultrasound (US) guidance would be non-inferior to fluoroscopy (FL) guidance for the primary endpoint of technical success at the C3–C6 levels (non-inferiority margin −5 percentage points), while offering shorter procedure time and eliminating radiation exposure. We further hypothesized that US and FL would yield comparable safety profiles and similar improvements in pain intensity (VAS) and functional status (NDI) over follow-up.

The findings of this single-center study are expected to provide valuable support for clinical decision-making by informing modality selection in settings where radiation minimization and workflow efficiency are critical. Moreover, this study is intended to the support broader adoption of radiation safety principles, such as the “As Low As Reasonably Achievable (ALARA)” standard, as well as the standardized reporting of radiation dose metrics, including fluoroscopy time, DAP, and air kerma, in FL-guided procedures [14,15].

## 2. Materials and Methods

### 2.1. Study Design and Setting

This single-center, observational, retrospective cohort study was conducted between March 2022 and July 2025 at the Adana City Training and Research Hospital, Department of Algology. Reporting was performed in accordance with the Strengthening the Reporting of Observational Studies in Epidemiology (STROBE) guidelines [16]. Safety events were graded using the Cardiovascular and Interventional Radiological Society of Europe (CIRSE) classification system [17]. The study protocol was approved by the Adana City Training and Research Hospital Clinical Research Ethics Committee (Decision No: 2026/1020, Date: 15 January 2026). Due to the retrospective nature of the study, the requirement for written informed consent was waived, in compliance with the Declaration of Helsinki and national regulations.

### 2.2. Participants

In total, 266 cervical medial branch procedures were screened. Forty-two cases were excluded due to acute neurological deficit (*n =* 6); local/systemic infection *(n* = 5); coagulopathy *(n* = 7); severe deformity or fusion (*n* = 8); additional procedures performed in the same session, such as epidural or foraminal injection or neuromodulation (*n* = 9); and protocol deviation or incomplete data (*n* = 7). The final analysis included 224 procedures (US-guided, *n* = 104; FL-guided, *n* = 120) (Supplementary Figure S1).

The inclusion criteria were

(i) Clinically suspected cervical facet-related pain characterized by segmental paravertebral pain, exacerbation with extension/rotation, and tenderness on facet provocation tests;
(ii) Radiological findings consistent with facet arthropathy or spondylosis at the C3–C6 levels;
(iii) Prior administration of US- or FL-guided medial branch block (MBB) and/or radiofrequency ablation (RFA).

The exclusion criteria included acute neurological deficits, cases with severe psychiatric illness, systemic or local infection, coagulopathy, severe spinal deformity or fusion preventing access, protocol deviations, and any additional epidural/foraminal injection or neuromodulation performed in the same session. Patients with severe spinal deformity, previous cervical spine surgery, instability, or severe spinal canal stenosis were excluded from the study.

Because this study has a retrospective design, patients were assigned to ultrasonography (US) or fluoroscopy (FL) groups not by a predetermined randomization method, but according to the imaging modality used in clinical practice. The choice of imaging method was made in accordance with clinical practice preferences, device accessibility, and the assessment of the practicing physician during the relevant period. Therefore, the comparison between the US and FL groups was carried out within an observational framework reflecting real-life practice.

### 2.3. Interventions and Procedures

All interventional procedures included in this study were performed by specialist physicians experienced in the field of algology. Fluoroscopy (FL) guided procedures were performed by an algology specialist with approximately 12 years of experience in cervical interventional pain procedures. Ultrasonography (US) guided procedures were performed by an algology specialist with approximately 4 years of experience in US-guided interventions. Both practitioners had received structured training in both US and FL-guided cervical medial branch interventions prior to the study period and regularly used these techniques in their clinical practice.

#### 2.3.1. Ultrasound (US)-Guided MBB/RFA

**Figure 1:**
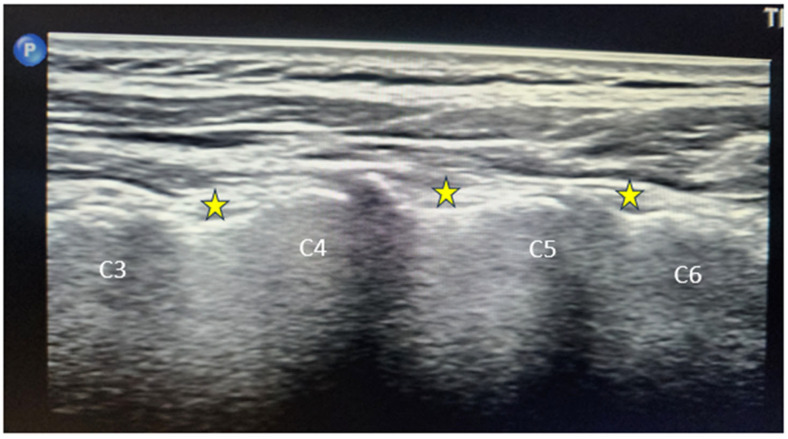
Long-axis ultrasound image of the cervical spine demonstrating the articular pillars at the C3–C6 levels. The cervical medial branch target points are marked with yellow stars at the waist of each articular pillar, corresponding to the typical anatomical location of the medial branches for diagnostic block or radiofrequency procedures.

All procedures were performed using a Philips Affiniti 70 ultrasound system (Philips Healthcare, Best, The Netherlands) equipped with musculoskeletal imaging presets and color/power Doppler capabilities, using a high-frequency linear-array transducer (10–15 MHz), a sterile probe cover, and sterile gel. A standard in-plane/long-axis approach was used to identify the lateral mass and medial branch grooves. (Figure 1) The needle types included 22G–25G, 50–100 mm, and 22G RF cannulas with 5–10 mm active tips for RFA. To prevent intravascular puncture, vascular structures were identified using power/color Doppler [17]. Verification consisted of real-time US visualization of the needle tip, aspiration testing, and optional sensory (≈50 Hz) and motor (≈2 Hz) stimulation during RFA. Local anesthetic injections consisted of 1–2% lidocaine (0.3–0.5 mL/ramus). If steroids were administered, their type and dose were recorded. The conventional RF parameters were 60–80 °C for 60–90 s, while pulsed RF was set at 42 °C for 240 s, following international standards [5].

#### 2.3.2. Fluoroscopy (FL)-Guided MBB/RFA

**Figure 2:**
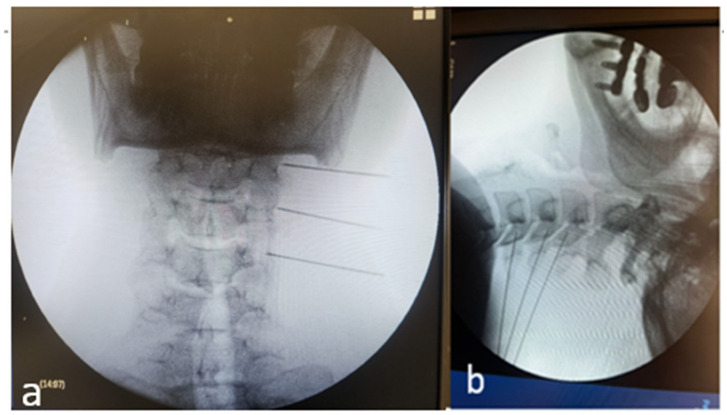
Fluoroscopy-guided cervical medial branch intervention. (**a**) Anteroposterior fluoroscopic view demonstrating alignment of the cervical vertebral bodies and identification of the target levels prior to needle advancement. (**b**) Oblique fluoroscopic view showing correct needle placement along the lateral aspect of the cervical articular pillars at the target levels (C3–C6), consistent with standard technique for cervical medial branch block or radiofrequency procedures.

Procedures were performed using a mobile C-arm with low-dose protocols, in line with the As Low As Reasonably Achievable (ALARA) principle. The lateral mass was targeted in the anteroposterior and lateral projections using osseous landmarks (Figure 2). The needle/RF equipment was identical to that used for US. Intravascular or intra-articular injection was excluded with iodinated contrast, followed by optional sensorimotor stimulation. Radiation exposure metrics—fluoroscopy time (min), dose–area product (DAP, Gy·cm^2^), and air kerma (mGy)—were recorded directly from console logs, and device calibration information was documented [8].

### 2.4. Common Procedure Elements

Standardized forms were used to collect data on sedation regimen (midazolam/fentanyl), laterality (unilateral/bilateral), treated level(s) (C3–C6), number of needle passes, injection volume, and total procedure duration (time from skin puncture to needle removal, measured with a stopwatch).

### 2.5. Sample Size and Power Analysis

Sample size estimation was based on the NI framework for the primary endpoint of technical success. Based on prior studies, the expected success rate was ≈90% for both modalities. An NI margin of −5 percentage points and a one-sided significance level of α = 0.025 were adopted, in line with CONSORT non-inferiority reporting recommendations [13]. Approximately 130–150 patients per group (≈260–300 total) were required for 80% power. With 224 patients (US = 104, FL = 120), the power achieved for the primary endpoint was ≈72%.

For clinical outcomes, exploratory MCID-based NI analyses were conducted with thresholds of 1.5 points for VAS and 7 points for NDI. Under typical standard deviations reported in the literature (VAS: 2.0–3.0; NDI: 9–12), statistical power ranged between 97 and 100% at 6 and 12 months [13,14].

### 2.6. Outcome Measures

Primary Endpoint:

- Technical success, defined as real-time confirmation of correct needle placement and independent verification by a blinded algology expert.

Secondary Endpoints:

- Procedure time (minutes).
- Pain intensity was assessed using a 10 cm Visual Analog Scale (VAS; 0 = no pain, 10 = worst imaginable pain) at baseline immediately prior to the procedure and at each follow-up visit. Pain intensity (VAS, 0–10) at 1, 3, 6, and 12 months. Responders were defined as demonstrating ≥2-point improvement.
- Functional outcomes, assessed using the NDI (0–50).
- Complications graded according to CIRSE classification.
- Radiation exposure in the FL group (fluoroscopy time, DAP, and air kerma).
- Sedation dosage and steroid use.

Exploratory Endpoint:

- MCID-based NI analyses at 6 and 12 months (margin: 1.5 points for VAS; 7 points for NDI) were applied in a post hoc framework.

### 2.7. Data Collection

Demographic, clinical, and procedural data were obtained from institutional electronic medical records. VAS and NDI scores were systematically collected at outpatient visits (at baseline and at 1, 3, 6, and 12 months). Missing data were evaluated and are reported in Supplementary Tables S1 and S2, and complete-case analysis was applied primarily.

### 2.8. Statistical Analysis

The analysis sets included both the full cohort and Inverse Probability of Treatment Weighting (IPTW) cohorts. Propensity score models included demographic and clinical covariates; covariate balance was evaluated using standardized mean difference (SMD) thresholds of <0.10, consistent with established recommendations [18,19].

Continuous outcomes (procedure time, VAS, and NDI) were analyzed using baseline-adjusted mixed-effects linear models with a random intercept and time × group interaction. Binary outcomes were analyzed with IPTW-supported mixed-effects logistic regression. Rare adverse events were reported as composite endpoints.

Clustering by operator was addressed with mixed-effects models and generalized estimating equations (GEE) to account for intra-patient correlation. Multiple sensitivity analyses were conducted, including per-protocol analyses, the exclusion of operator learning periods, alternative weighting/matching, Winsorization for outliers, and multiple imputation for missing data. Bayesian complementary NI analysis was performed to assess the posterior probability of NI under weak priors, in line with best practice for robustness [20].

## 3. Results

### 3.1. Baseline Characteristics

A total of 266 procedures were screened, of which 42 were excluded (acute neurological deficit, *n* = 6; local/systemic infection, *n* = 5; coagulopathy, *n* = 7; severe deformity/fusion, *n* = 8; additional procedures in the same session, *n* = 9; protocol deviation/incomplete data, *n* = 7). The final cohort comprised 224 procedures (US, *n* = 104; FL, *n* = 120) (Supplementary Table S1, Supplementary Figure S1). Weighted IPTW results are reported exploratorily (Supplementary Table S2, Supplementary Figure S2). Missing data summaries are provided in Supplementary Materials, and the primary analyses were conducted using a complete-case approach.

Baseline characteristics were comparable across groups in terms of age, sex, Body Mass Index (BMI), target cervical levels (C3–C6), and procedure type (Table 1). The mean age was 57.3 ± 9.7 years in the US group and 56.9 ± 10.4 years in the FL group; the mean BMI was 27.7 ± 4.3 and 27.0 ± 4.9 kg/m^2^ (*p* = 0.24), respectively. Female patients constituted 51.9% of the US group and 55.0% of the FL group. Target-level distributions (US vs. FL) were as follows: C3, 33.7% vs. 35.8%; C4, 42.3% vs. 46.7%; C5, 35.6% vs. 39.2%; and C6, 39.4% vs. 34.2%.

### 3.2. Pain and Functional Outcomes

**Figure 3:**
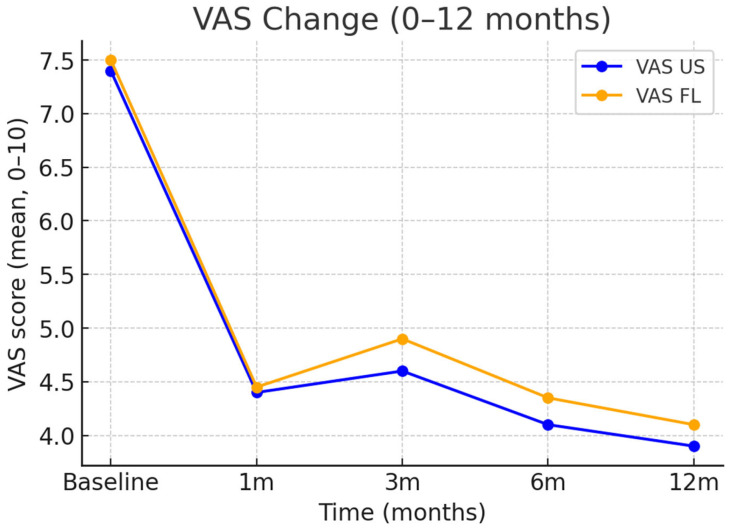
Longitudinal changes in pain intensity (VAS) in US- and FL-guided groups. Line graph showing mean Visual Analog Scale (VAS) scores from baseline to 12 months for patients undergoing ultrasound (US)-guided and fluoroscopy (FL)-guided cervical medial branch procedures. Dots represent mean value at each time point (baseline, 1, 3, 6, and 12 months); lines indicate temporal trends. Error bars show standard deviation (SD). Abbreviations: VAS = Visual Analog Scale; US = Ultrasound; FL = Fluoroscopy; SD = Standard Deviation. Units: VAS score (0–10).

**Figure 4:**
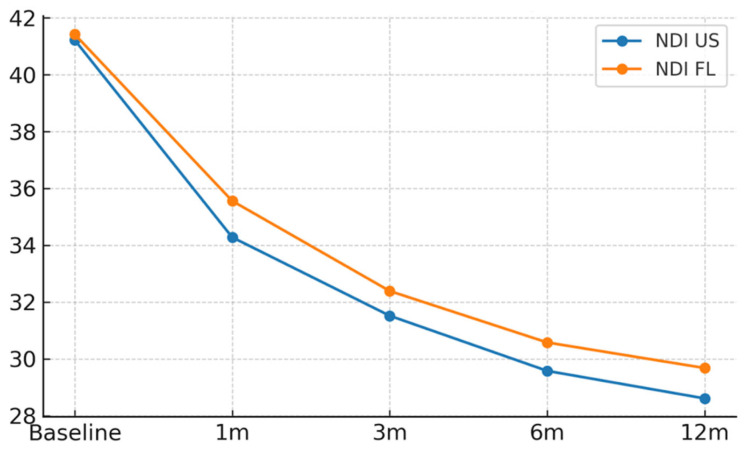
Longitudinal changes in functional status (NDI) in US- and FL-guided groups. Line graph showing mean Neck Disability Index (NDI) scores from baseline to 12 months for patients undergoing US- and FL-guided procedures. Dots represent mean value at each time point (baseline, 1, 3, 6, and 12 months); lines indicate temporal trends. Error bars show SD. Abbreviations: NDI = Neck Disability Index; US = Ultrasound; FL = Fluoroscopy; SD = Standard Deviation. Units: NDI score (0–50).

At baseline (pre-procedure), VAS scores were similar between groups (US: 7.31 vs. fluoroscopy: 7.47), with a mean between-group difference of −0.16 points. Baseline VAS (pre-procedure) values were recorded for all patients and are presented in Table 1. At three months, the average decrease in Visual Analog Scale (VAS) scores was 2.7 points in the US group and 2.6 points in the FL group (difference: 0.1; 95% CI: −0.3 to 0.5), with the groups experiencing improvements in the Neck Disability Index (NDI) of 9.7 and 9.0 points, respectively (difference: 0.7; 95% CI: −1.0 to 2.3). The responder rate (≥2-point improvement in VAS) was 66.3% for US and 65.8% for FL (difference: 0.5 percentage points; 95% CI: −11.9 to 12.9) (Table 2, Figure 3 and Figure 4).

### 3.3. Secondary Endpoint: Procedure Time

**Figure 5:**
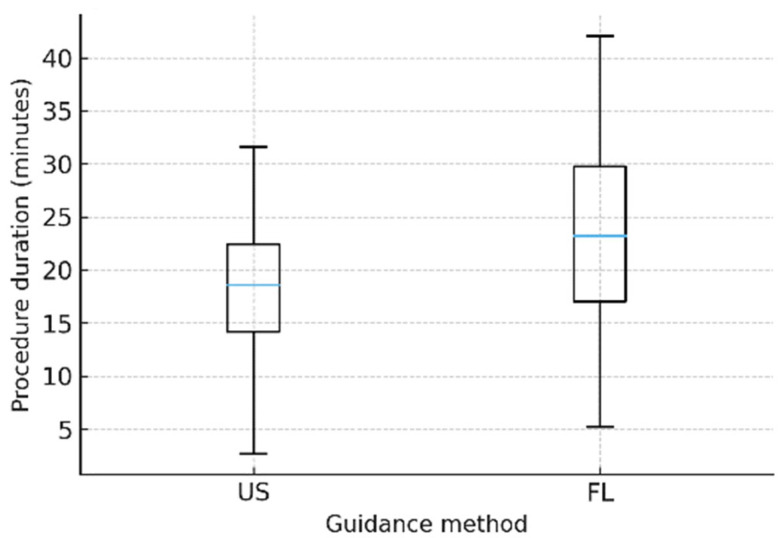
Procedure time distribution (US vs. FL). Box plot showing procedure time distribution in minutes. The central line indicates the median; the lower and upper box boundaries indicate the interquartile range (Q1–Q3); and whiskers indicate the range up to 1.5 × IQR. Outliers are displayed as individual points. Figure Abbreviations: US = ultrasound; FL = fluoroscopy; IQR = interquartile range. Units: procedure time (minutes).

The mean procedure time was significantly shorter in the US group (18.1 ± 7.6 min; median 19.0, IQR 12.3–23.4) compared with the FL group (23.3 ± 9.9 min; median 22.7, IQR 15.2–29.4). The mean difference (US–FL) was −5.2 min (95% CI: −7.5 to −2.9), a difference both statistically significant and clinically relevant (Table 3 and Figure 5).

Longitudinal analysis showed that VAS scores decreased from a baseline of 7.39 to 3.96 at 12 months (US group) and from 7.47 to 3.98 (FL group). The NDI scores improved from 41.3 at baseline to 29.2 at 12 months, with no meaningful differences between groups. These findings indicate sustained and comparable improvements in both modalities (Table 2, Figure 3 and Figure 4).

### 3.4. Primary Endpoint: Technical Success

**Figure 6:**
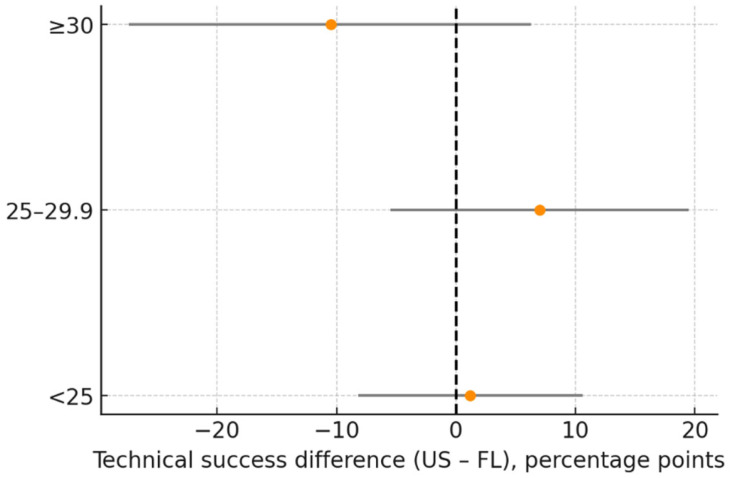
Technical success difference (US–FL) in BMI subgroups. Forest plot of subgroup analysis. Dots represent the effect estimates, horizontal lines indicate the 95% Confidence Interval (CI), and the vertical reference line indicates the neutral (0) effect. Subgroups analyzed: BMI < 25; BMI 25–29.9; BMI ≥ 30. Abbreviations: US = ultrasound; FL = fluoroscopy; BMI = Body Mass Index; CI = confidence Interval. Units: percentage points.

The technical success rate was 90.4% in the US group and 90.8% in the FL group, with an absolute difference of −0.4 percentage points (95% CI: −8.1 to 7.2). The predefined non-inferiority (NI) margin was set at −5 percentage points, with a one-sided α = 0.025. Since the lower limit of the confidence interval (−8.1) fell below this margin, statistical non-inferiority was not established (Table 4, Figure 6). This suggests that, while the observed rates were clinically similar, the limited sample size and wide confidence interval prevented the formal confirmation of NI.

### 3.5. Exploratory NI Analyses of Clinical Outcomes

Post hoc analyses using MCID-based NI margins (1.5 points for VAS, 7 points for NDI) demonstrated high statistical power (97–100%) at 6 and 12 months, even under varying standard deviations (VAS: 2.0–3.0; NDI: 9–12). The small between-group differences further supported clinical equivalence. Sample-size calculations indicated that only 44 patients were needed for 80% power at a 1.5-point VAS margin and 33 patients for an NDI margin of 7 points, suggesting that the current sample (US = 104, FL = 120) was more than sufficient for an exploratory NI analysis.

### 3.6. Sedation and Steroid Use

The mean sedation dose was 1.6 ± 0.5 mg of midazolam and 42 ± 10 µg of fentanyl in the US group and 1.7 ± 0.6 mg of midazolam and 45 ± 12 µg of fentanyl in the FL group, with no significant differences (*p* > 0.05). Steroid use was comparable (US 18% vs. FL 21%, *p* = 0.64). Radiofrequency ablation (RFA) parameters (80–90 °C, 60–90 s) were homogeneously distributed. Pulsed RF was applied in 6% of cases—too few for separate statistical analysis.

### 3.7. Safety Outcomes

**Figure 7:**
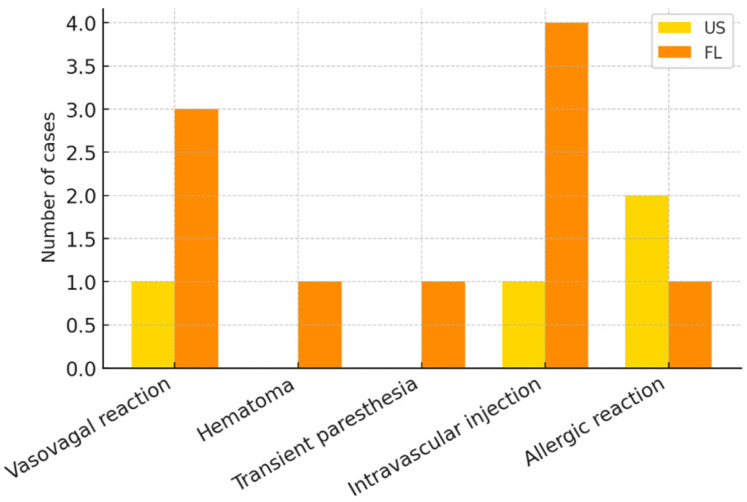
Complication profile (US vs. FL). Bar chart of complications classified according to the Cardiovascular and Interventional Radiological Society of Europe (CIRSE) criteria. Bar heights indicate the number of observed events. No major complications were observed. Abbreviations: US = ultrasound; FL = fluoroscopy; CIRSE = Cardiovascular and Interventional Radiological Society of Europe. Units: number of cases.

According to the CIRSE classification, complication rates were 3.8% in the US group and 7.5% in the FL group (OR: 0.49; 95% CI: 0.15–1.65). No major complications were observed. The complication spectrum is summarized in Figure 7.

### 3.8. Radiation Exposure (FL Group)

In the FL group (*n* = 120), the mean fluoroscopy time was 2.28 min (median: 2.34, IQR: 1.66–2.94), the mean DAP was 4.08 Gy·cm^2^ (median: 4.18, IQR: 2.83–5.48), and the mean air kerma was 37.5 mGy (median: 38.9, IQR: 25.5–47.2). These values confirm adherence to ALARA (As Low As Reasonably Achievable) standards (Table 5).

### 3.9. Subgroup Analyses

**Figure 8:**
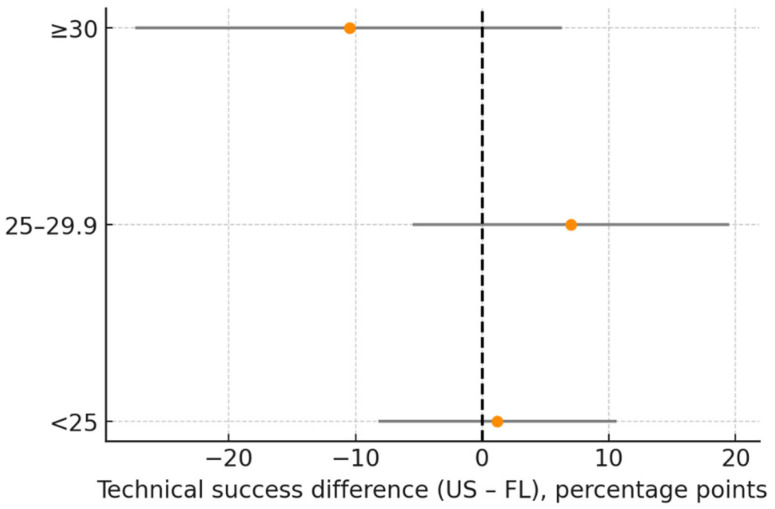
Technical success difference (US–FL) by gender. Forest plot of subgroup analysis. Dots represent the effect estimates, horizontal lines indicate the 95% CI, and the vertical reference line indicates the neutral (0) effect. Gender subgroups: female; male. Abbreviations: US = ultrasound; FL = fluoroscopy; CI = confidence Interval. Units: percentage points.

Gender: The difference in technical success (US–FL) was 1.3 percentage points (95% CI: −9.4 to 12.1) in women and −2.6 points (95% CI: −13.5 to 8.3) in men (p for interaction = 0.608; Table 4, Figure 8).

**Figure 9:**
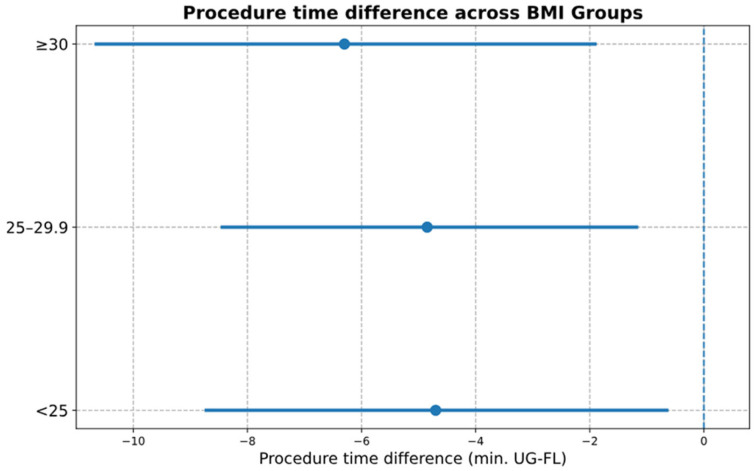
Difference in procedure time (US–FL) in BMI subgroups. Forest plot showing mean differences in procedure time (minutes) between US and FL guidance across BMI categories. Dots represent mean difference; horizontal lines represent 95% CI; the vertical reference line indicates the neutral (0) effect. Abbreviations: US = ultrasound; FL = fluoroscopy; BMI = Body Mass Index; CI = confidence Interval. Units: Minutes.

**Figure 10:**
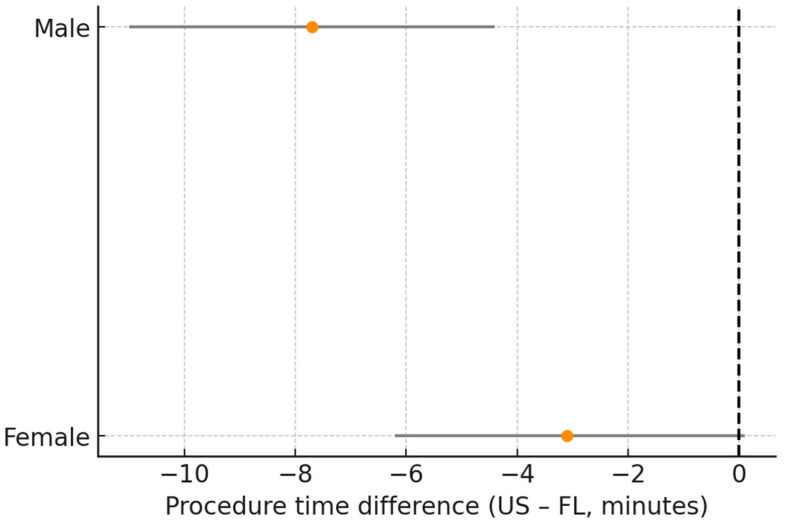
Difference in procedure time (US–FL) by gender. Forest plot of subgroup analysis for gender. Dots represent mean differences in procedure time (minutes); horizontal lines represent 95% CI; the vertical line indicates the neutral (0) effect. Abbreviations: US = ultrasound; FL = fluoroscopy; CI = confidence interval. Units: Minutes.

**Figure 11:**
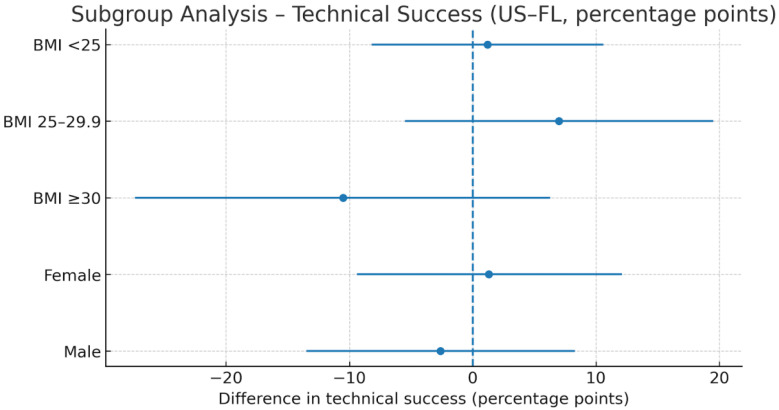
Subgroup analysis of technical success (US–FL difference, percentage points). Forest plot showing technical success differences expressed in percentage points. Dots represent mean difference; horizontal lines represent 95% CI; vertical dashed line indicates neutral effect (0). Abbreviations: US = ultrasound; FL = fluoroscopy; CI = confidence interval. Units: percentage points.

**Figure 12:**
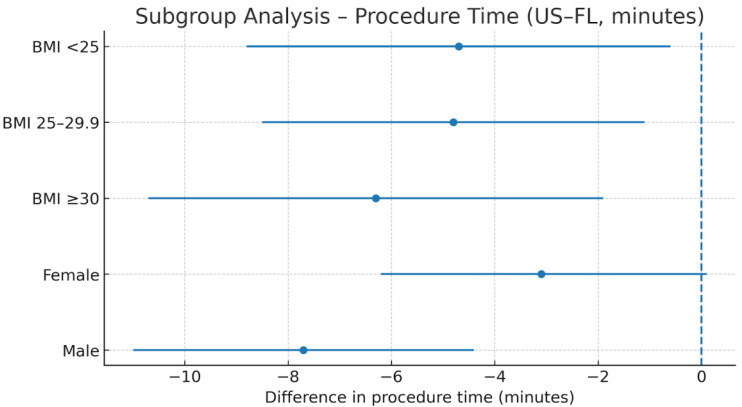
Subgroup analysis of procedure time (US–FL difference, minutes). Forest plot of subgroup analysis of procedure time. Dots represent mean difference (minutes); horizontal lines represent 95% CI; vertical dashed line indicates neutral effect (0). Abbreviations: US = ultrasound; FL = fluoroscopy; CI = confidence interval. Units: Minutes.

BMI and Procedure Time: Differences in procedure time (US–FL) were −4.7 min (95% CI: −8.8 to −0.6) for BMI < 25, −4.8 min (95% CI: −8.5 to −1.1) for BMI 25–29.9, and −6.3 min (95% CI: −10.7 to −1.9) for BMI ≥ 30. No significant interaction was observed (*p* = 0.851; Table 5, Figure 9, Figure 10, Figure 11 and Figure 12).

## 4. Discussion

This single-center cohort compared ultrasound (US) and fluoroscopy (FL) guidance for cervical medial branch procedures (C3–C6) across technical success, procedure time, clinical outcomes, safety, and radiation exposure. Technical success rates were high and closely matched between modalities; the predefined non-inferiority (NI) margin was not met, but the absolute difference (−0.4 percentage points) was small and unlikely to be clinically meaningful. US conferred a clear efficiency advantage, with significantly shorter procedure times, and eliminated radiation exposure, supporting patient and staff safety.

Our findings align with randomized trials showing comparable performance between US and FL in the cervical region. In a randomized trial of C7 medial branch blocks (*n* = 50), Finlayson et al. found no difference between US and FL [21]; a second randomized study on third occipital nerve blocks (*n* = 40) reported similar results [5]. A retrospective comparative study of cervical medial branch blocks *(n* = 126) also reported similar clinical responses, with shorter procedure times under US [22]. In combination with the present data, these studies indicate that US achieves technical and clinical outcomes comparable to FL while improving workflow efficiency.

In our cohort, improvements in pain (VAS) and function (NDI) were significant and sustained over 12 months, with no clinically important between-group differences. Exploratory MCID-based NI analyses (margins: 1.5 points for VAS; 7 points for NDI) were highly powered and favored the clinical non-inferiority of US, reinforcing practical equivalence over the follow-up horizon. Because technical success was the prespecified primary endpoint, these clinical NI findings should be viewed as hypothesis-generating.

No major complications were observed in either treatment arm, in alignment with the findings of previous studies. In a recent systematic review and meta-analysis, Viderman et al. (2023) reported that ultrasound-guided spinal and facet interventions were associated with a similarly low rate of major complications while demonstrating a significantly reduced risk of vascular injury compared with fluoroscopy-guided techniques [23]. Finlayson et al. evaluated approximately 120 ultrasound-guided cervical medial branch block procedures performed in 60 patients and reported only minor transient adverse events, emphasizing that real-time visualization of vascular structures under ultrasound significantly enhances safety and reduces the likelihood of inadvertent vascular puncture [24]. Our adverse-event reporting used the CIRSE system; future studies may complement this with Clavien–Dindo classification to harmonize grading across interventional disciplines [18,25].

FL-arm dose metrics (fluoroscopy time, DAP, and air kerma) fell within the ranges reported in the prospective and methodological literature, reflecting adherence to ALARA principles [14,16]. The absence of ionizing radiation with US remains a compelling advantage, particularly for repeat or multi-level interventions and high-throughput ambulatory settings.

The US time advantage was consistent across BMI strata and genders in our data. Prior work supports the feasibility of US in challenging habitus: Rauch et al. reported successful US-guided medial branch blocks in 84 obese patients [26]. A CUSUM-based learning-curve analysis further suggests that procedure times decrease with more US experience (sample size not reported) [27]. These observations, together with guideline perspectives from a multispecialty panel (≈30 experts), support tailoring modality choice to operator skill, case complexity, and safety priorities [5].

PRF has been suggested as a theoretically safer option because it provides neuromodulatory effects without creating thermal destruction in the target tissue. However, PRF application was not preferred in our current study. The main reason for this preference is that conventional radiofrequency (RF) ablation provides a longer-lasting clinical effect compared to PRF. Studies in the literature that directly compare RF and PRF approaches show that RF offers more lasting results in terms of pain control. Tekin et al., in their randomized controlled trial involving 60 patients with facet-related chronic low back pain, reported that the significant improvement in pain scores in the conventional RF group lasted until the 12th month, while the effectiveness was shorter in the PRF group [28]. Similarly, Van Zundert et al., in their randomized controlled trial in 40 patients with cervical pain, showed that PRF provided short- and medium-term pain reduction but was not superior to conventional RF in terms of long-term effectiveness [29]. In this context, considering the long-term pain control and functional gains targeted in our study, the conventional RF approach was preferred over PRF. However, it should be acknowledged that PRF may be a potential option in selected patient groups where tissue destruction is undesirable or where the neuropathic component is prominent. The clinical place and long-term effectiveness of PRF in cervical medial branch interventions should be more clearly demonstrated with well-designed prospective comparative studies in the future.

From a practical perspective, ultrasound (US) and fluoroscopy (FL) guidance each have distinct advantages and trade-offs for cervical medial branch interventions. US provides real-time visualization of superficial soft tissues and adjacent vascular structures and, critically, eliminates ionizing radiation exposure for both patients and staff [5]. In contrast, FL offers highly standardized bony landmark visualization with well-established workflows, but inherently exposes patients and operators to ionizing radiation, the magnitude of which is closely related to fluoroscopy time and procedural factors [14]. In our cohort, US guidance was associated with a significantly shorter procedure time (mean difference −5.2 min; *p* < 0.001), while FL required an average fluoroscopy time of 2.28 min (with low radiation doses). These findings are consistent with prior comparative work in cervical medial branch blocks, where US guidance achieved similar clinical outcomes with shorter administration times and fewer needle passes compared with FL. Recent reviews further support that US-guided axial facet/medial branch interventions can achieve favorable accuracy with reduced procedural time, while emphasizing that image quality and landmark identification may be more challenging in patients with high BMI or complex anatomy [22]. Beyond procedural efficiency, radiation exposure is an important differentiator: although per-procedure doses during spinal interventions can be relatively low, cumulative occupational exposure remains a recognized concern for interventional clinicians, supporting efforts to minimize fluoroscopy time and optimize radiation safety practices [14]. With respect to costs, direct cost comparisons are highly dependent on local reimbursement and institutional accounting. Because itemized cost/billing data were not captured in this retrospective dataset, we did not perform a formal cost-effectiveness analysis. Nevertheless, US may reduce resource utilization by avoiding the need for a fluoroscopy suite, radiation shielding, and radiographic equipment operation/maintenance, whereas FL requires dedicated imaging infrastructure and radiation-protection workflows [30]. Finally, our findings should be interpreted in light of potential unmeasured confounding: we did not systematically collect or adjudicate comorbidities and conditions that can influence neck pain and outcomes (e.g., extent of degenerative disease, neurological or mental health disorders, diabetes, hypertension). Therefore, residual confounding cannot be excluded, and future prospective studies should incorporate standardized assessment of these factors and include formal economic evaluation alongside clinical outcomes [5].

Detailed ROC metrics (e.g., AUC values, alternative cut-offs, and diagnostic indices such as PPV/NPV, and likelihood ratios) are provided in the Supplementary Materials to avoid over-technical emphasis in the main text. Briefly, while certain cut-offs showed excellent discrimination in this dataset, external validation in prospective cohorts is needed prior to clinical adoption.

Given the comparable technical and clinical effectiveness, the absence of radiation and shorter procedure times justify considering US as a first-line guidance option when operator expertise is available, particularly in outpatients and radiation-sensitive populations. FL remains valuable for complex anatomies (e.g., severe deformity, multi-level work, revision cases) where bony detail and multiplanar confirmation are paramount.

## 5. Limitations

This study has several limitations. Due to its retrospective nature, patients were not randomly assigned to ultrasound (US) or fluoroscopy (FL) guidance; this could lead to selection bias and indication-related confusion. In addition, not all patients underwent the same intervention (radiofrequency ablation [RFA] and/or diagnostic/therapeutic block), and the decision to perform a block or RFA may be influenced by symptom severity, chronicity, or previous treatment response. In our cohort, the distribution of procedure type was generally comparable among imaging groups—US: 48/104 (46.2%) RFA, 34/104 (32.7%) diagnostic block, 22/104 (21.2%) therapeutic block; FL: 50/120 (41.7%) RFA, 44/120 (36.7%) diagnostic block, 26/120 (21.7%) therapeutic block. This reduces, but does not completely eliminate, the potential confounding effect of treatment heterogeneity. Furthermore, outcome assessment relied on routinely documented measurements and follow-up schedules, therefore measurement bias and missing data/missing data bias cannot be entirely ruled out. Specifically, we did not systematically capture or adjudicate several conditions that may contribute to neck pain and influence outcomes, such as the severity of degenerative changes on imaging, neurological disorders, mental health conditions (e.g., anxiety/depression), or comorbidities such as diabetes and hypertension. These factors may affect baseline symptom burden, pain perception, and response to intervention; therefore, residual confounding cannot be excluded. Prospective studies incorporating standardized assessment of comorbidities and imaging severity (and, ideally, randomized allocation of imaging guidance) are warranted. Finally, due to its single-center nature, generalizability to other settings may be limited. Prospective studies with standardized intervention layers (block only vs. RFA only) and predefined follow-up schedules are needed. Prospective multicenter NI/equivalence trials with prespecified clinical endpoints (responder status, composite safety), standardized reporting (CIRSE/Clavien–Dindo), HRQoL/cost-effectiveness measures, and ≥12-month follow-up at C3–C6 are warranted.

## 6. Conclusions

In this single-center observational study, ultrasound (US) and fluoroscopy (FL) guidance achieved high technical success rates in cervical medial branch procedures at the C3–C6 levels. The primary endpoint, technical success, did not meet the predefined non-inferiority (NI) margin due to the limited sample size (difference: −0.4 percentage points; 95% CI: −8.1 to 7.2). This finding should be regarded as statistically inconclusive rather than clinically meaningful, and exploratory NI analyses are hypothesis-generating only.

US provided practical advantages, including a significantly shorter procedure time and the complete elimination of radiation exposure, while maintaining a safety profile comparable to FL. Both short-term (1–3 months) and long-term (6–12 months) follow-ups demonstrated sustained improvements in pain intensity (VAS) and functional outcomes (NDI) across both modalities. Improvements in NDI may also be considered proxies for health-related quality of life (HRQoL), although standardized HRQoL instruments (e.g., EQ-5D, SF-36) were not applied in this study. Exploratory ROC findings indicated potential diagnostic value but remain sample-specific and require independent prospective validation.

Clinically, US appears to be a safe and effective alternative, particularly in centers prioritizing radiation reduction and workflow efficiency, while FL remains valuable in complex multi-level or anatomically challenging cases. Future multicenter prospective NI or equivalence trials with ≥12 months of follow-up are needed to confirm these findings. Such trials should also incorporate standardized HRQoL and cost-effectiveness assessments to inform evidence-based clinical guidelines.

## Figures and Tables

**Table 1:** Baseline demographic and clinical characteristics.

Characteristic	US (*n* = 104)	FL (*n* = 120)
Age (years), mean ± SD	57.3 ± 9.7	56.9 ± 10.4
BMI (kg/m^2^), mean ± SD	27.7 ± 4.3	27.0 ± 4.9
Female, *n* (%)	54 (51.9)	66 (55.0)
Target level: C3, *n* (%)	35 (33.7)	43 (35.8)
Target level: C4, *n* (%)	44 (42.3)	56 (46.7)
Target level: C5, *n* (%)	37 (35.6)	47 (39.2)
Target level: C6, *n* (%)	41 (39.4)	41 (34.2)
Procedure: RFA, *n* (%)	48 (46.2)	50 (41.7)
Procedure: Diagnostic block, *n* (%)	34 (32.7)	44 (36.7)
Procedure: Therapeutic block, *n* (%)	22 (21.2)	26 (21.7)

Legend. Data are presented as the mean ± standard deviation (SD) or number (%). BMI = Body Mass Index; RFA = Radiofrequency Ablation; C3–C6 = cervical spinal levels.

**Table 2:** Primary and secondary outcomes.

Outcome	Timepoint	US (Mean)	FL (Mean)	Difference (US–FL)
VAS	Baseline	7.31	7.47	−0.16
NDI	Baseline	41.22	41.42	−0.20
VAS	1 month	4.40	4.45	−0.05
NDI	1 month	34.28	35.56	−1.28
VAS	3 months	4.62	4.87	−0.25
NDI	3 months	31.52	32.39	−0.87
VAS	6 months	4.09	4.35	−0.26
NDI	6 months	29.58	30.58	−1.00
VAS	12 months	3.82	4.09	−0.27
NDI	12 months	28.61	29.68	−1.07

Legend. Values represent mean scores for US- and FL-guided groups from baseline to 12 months. VAS = Visual Analog Scale; NDI = Neck Disability Index. Differences are crude (unadjusted).

**Table 3:** Subgroup analysis: procedure time (US–FL difference).

Subgroup	US–FL Difference (min)	95% CI	Interaction *p*	Comment
BMI < 25	−4.7	−8.8 to −0.6	0.851	Exploratory; not significant
BMI 25–29.9	−4.8	−8.5 to −1.1	-	-
BMI ≥ 30	−6.3	−10.7 to −1.9	-	-
Female	−3.1	−6.2 to 0.1	0.049	Exploratory; not significant after adjustment
Male	−7.7	−11.0 to −4.4	-	-

Legend. Differences are reported in minutes (US–FL). CI = Confidence Interval; BMI = Body Mass Index. *p* values are exploratory due to multiple comparisons.

**Table 4:** Subgroup analysis: technical success (US–FL difference).

Subgroup	US–FL Difference (%)	95% CI	Interaction *p*	Comment
BMI < 25	+1.2	−8.2 to 10.6	0.267	Exploratory, not significant
BMI 25–29.9	+7.0	−5.5 to 19.5	-	-
BMI ≥ 30	−10.5	−27.4 to 6.3	-	-
Female	+1.3	−9.4 to 12.1	0.608	Exploratory, not significant
Male	−2.6	−13.5 to 8.3	-	-

Legend. Differences are reported as percentage points (US–FL). CI = Confidence Interval; BMI = Body Mass Index. *p* values are exploratory due to multiple comparisons.

**Table 5:** Radiation metrics in FL arm (*n* = 120).

Metric	Mean ± SD	Median (IQR)
Fluoroscopy time (min)	2.28	2.34 (1.66–2.94)
DAP (Gy·cm^2^)	4.08	4.18 (2.83–5.48)
Air kerma (mGy)	37.5	38.9 (25.5–47.2)

Legend. DAP = Dose–Area Product; IQR = Interquartile Range; SD = Standard Deviation.

## Data Availability

The data supporting the findings of this study are available from the corresponding author upon reasonable request. Due to institutional regulations, the raw data cannot be made publicly available.

## Supplementary Materials

The following supporting information can be downloaded at: https://www.mdpi.com/article/10.3390/diagnostics16040592/s1, Table S1. Patient screening and exclusion criteria; Table S2. IPTW-weighted outcomes (US vs. FL); Figure S1. Patient flow diagram; Figure S2. Covariate balance—Standardized Mean Difference.

## Abbreviations

US	Ultrasound
FL	Fluoroscopy
NI	Non-Inferiority
VAS	Visual Analog Scale
NDI	Neck Disability Index
RFA	Radiofrequency Ablation
MCID	Minimum Clinically Important Difference
IPTW	Inverse Probability of Treatment Weighting
SMD	Standardized Mean Difference
CIRSE	Cardiovascular and Interventional Radiological Society of Europe (complication classification)
ALARA	As Low As Reasonably Achievable (radiation safety principle)
ROC	Receiver Operating Characteristic
AUC	Area Under the Curve
HRQoL	Health-Related Quality of Life
BMI	Body Mass Index
DAP	Dose–Area Product
GEE	Generalized Estimating Equations
