# Supplementary material for: Ultrasound Versus Fluoroscopy for Cervical Medial Branch Injections at C3–C6: A Single-Center Retrospective Cohort Study
Source: Diagnostics (Basel). 2026 Feb 16;16(4):592. doi: 10.3390/diagnostics16040592 (PMC12939812; doi:10.3390/diagnostics16040592)
Supplement: Supplementary file 1 [file diagnostics-16-00592-s001.zip › diagnostics-4135427-supplementary.pdf]

Table S1. Patient screening and exclusion criteria.

Stage	Description	*n*
Screened	Total cervical medial branch procedures (Jan 2021—Dec 2024)	266
Excluded (total)	Did not meet inclusion criteria/met exclusion criteria	42
Acute neurological deficit	New/progressive neurological deficit	6
Local/systemic infection	Evidence of infection (spondylodiscitis; systemic infection; local site infection)	5
Coagulopathy	Coagulopathy or anticoagulant therapy contraindicating intervention	7
Severe deformity/fusion	Anatomical barriers preventing safe access	8
Additional procedures	Concurrent epidural/foraminal injection or neuromodulation in same session	9
Protocol deviation/incomplete data	Records incomplete or outside prespecified protocol	7
Included	Final cohort included in analysis	224
Allocation—US	Ultrasound-guided procedures	104
Allocation—FL	Fluoroscopy-guided procedures	120

Final cohort included 224 patients who underwent cervical medial branch procedures (US = 104; FL = 120) at Adana City Training and Research Hospital. Legend. Patient screening and exclusion consistent with STROBE guidelines. Reasons for exclusion are shown with case counts; total excluded = 42. Abbreviations: US—Ultrasound; FL—Fluoroscopy; STROBE—Strengthening the Reporting of Observational Studies in Epidemiology.

Table S2. IPTW-weighted outcomes (US vs. FL).

Outcome	US (IPTW)	FL (IPTW)	Difference (US–FL)	95% CI
Technical success (%)	90.2	90.6	−0.5	−8.4 to 7.4
Procedure time (minutes)	18.4	23.2	−4.8	−7.1 to −2.5
Any complication (%)	3.5	7.4	−3.8	−9.8 to 2.2
VAS change (0–3 months)	−2.9	−2.7	−0.2	−0.4 to 0.3
NDI change (0–3 months)	9.5	8.9	0.5	−1.2 to 2.1
Responder ≥2 VAS points (%)	63	67	−3.7	−16.4 to 9.0

LIPTW = Inverse Probability of Treatment Weighting; VAS = Visual Analog Scale (0–10); NDI = Neck Disability Index (0–50); CI = Confidence Interval. Differences expressed as US–FL. Analyses are exploratory/post hoc. Final cohort included 224 procedures (US = 104, FL = 120) after exclusion of 42 from 266 screened.

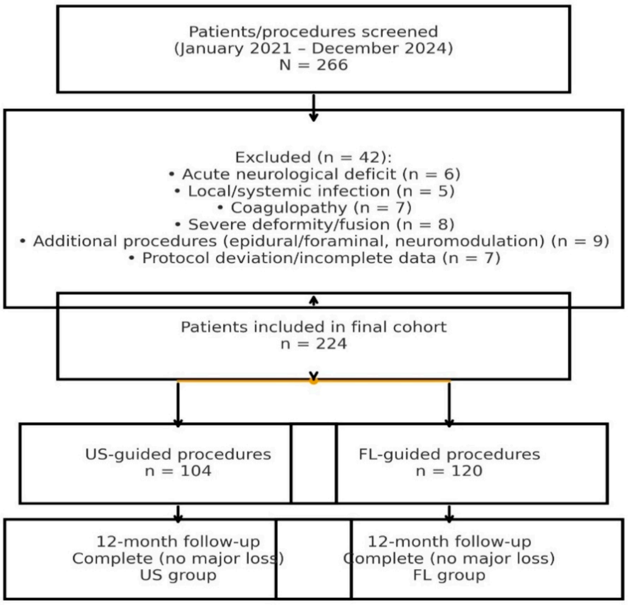

Figure S1. Patient flow diagram. Patient flow diagram showing case selection, exclusion criteria, and group allocation in accordance with the STROBE guidelines. A total of 224 procedures were included in the final analysis after applying predefined inclusion and exclusion criteria at Adana City Training and Research Hospital. Patients were stratified into the Ultrasound (US)-guided group (n = 104) or the Fluoroscopy (FL)-guided group (n = 120). All patients were followed for 12 months; no major loss to follow-up occurred. Abbreviations: US—Ultrasound; FL—Fluoroscopy; STROBE—Strengthening the Reporting of Observational Studies in Epidemiology.

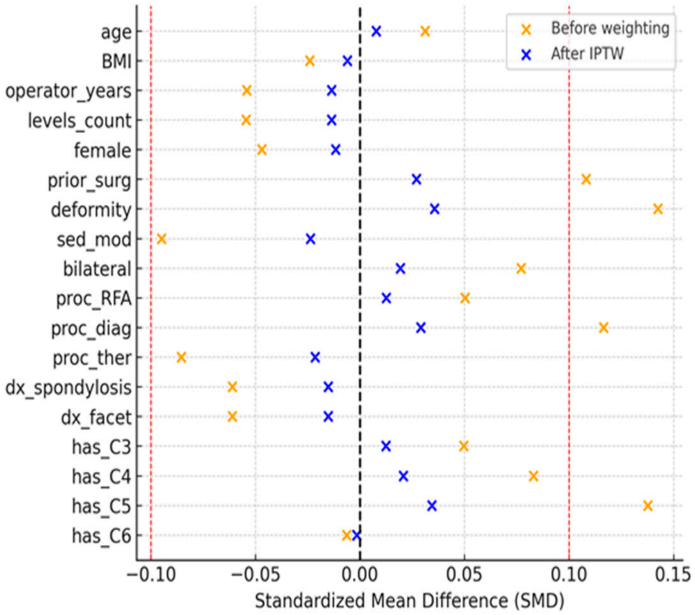

Figure S2. Covariate balance—Standardized Mean Difference. Scatter plot showing the Standardized Mean Differences (SMDs) for baseline covariates before and after Inverse Probability of Treatment Weighting (IPTW). Each dot represents a covariate imbalance estimate. The vertical dashed line at 0 indicates no imbalance; the red dashed lines at ±0.10 indicate the commonly accepted balance threshold. Abbreviations: SMD = standardized mean difference; IPTW = Inverse Probability of Treatment Weighting; proc_RFA = procedure: radiofrequency ablation; proc_diag = procedure: diagnostic block; proc_ther = procedure: therapeutic block; sed_mod = moderate sedation; bilateral = bilateral procedure; prior_surg = prior surgery; dx_facet = facet joint diagnosis; dx_spondylosis = spondylosis diagnosis; has_C3–C6 = target cervical level.
